# Supplementary material for: Polyketide synthase gene clusters in the ericoid mycorrhizal fungus Oidiodendron maius: genome-wide prediction, expression profiling, and functional characterization of knockout mutants
Source: Front Plant Sci. 2026 Sep 16;17:1924430. doi: 10.3389/fpls.2026.1924430 (PMC13623903; doi:10.3389/fpls.2026.1924430)
Supplement: Supplementary file 1 [file SupplementaryFile1.zip › Supplementary Materials/Supplementary Folder 1.zip!/Oidma1_AssemblyScaffolds_Repeatmasked/knownclusterblast/cluster50/jgi.p_Oidma1_45990_mibig_hits.html]

| MIBiG Protein | Description | MIBiG Cluster | MiBiG Product | % ID | % Coverage | BLAST Score | E-value |
| --- | --- | --- | --- | --- | --- | --- | --- |
| ADY16687.1 | TqaC | BGC0001142 | Tryptoquialanine\_biosynthetic\_gene\_cluster | 33 | 90.0537634409 | 141 | 1.5e-33 |
| BAJ09787.1 | dehydrogenase | BGC0000146 | Solanapyrone\_biosynthetic\_gene\_cluster | 37 | 68.8172043011 | 121 | 1.6e-27 |
| BAO66515.1 | retinol\_dehydrogenase | BGC0000042 | Cremimycin\_biosynthetic\_gene\_cluster | 26 | 77.4193548387 | 60 | 4.4e-09 |
